# Butyrophilins: Dynamic Regulators of Protective T Cell Immunity in Cancer

**DOI:** 10.3390/ijms24108722

**Published:** 2023-05-13

**Authors:** Rinkee Kumari, Elaheh Sadat Hosseini, Kristen E. Warrington, Tyler Milonas, Kyle K. Payne

**Affiliations:** 1 Medical Immunology, Rutgers Cancer Institute of New Jersey, New Brunswick, NJ 08901, USA; 2 Cellular and Molecular Pharmacology, Rutgers School of Graduate Studies, Rutgers, The State University of New Jersey, New Brunswick, NJ 08854, USA; 3 Department of Medicine, Robert Wood Johnson Medical School, Rutgers, The State University of New Jersey, New Brunswick, NJ 08901, USA

**Keywords:** immune oncology, immunotherapy, immune suppression, butyrophilins, γδ T cells

## Abstract

The efficacy of current immunotherapies remains limited in many solid epithelial malignancies. Recent investigations into the biology of butyrophilin (BTN) and butyrophilin-like (BTNL) molecules, however, suggest these molecules are potent immunosuppressors of antigen-specific protective T cell activity in tumor beds. BTN and BTNL molecules also associate with each other dynamically on cellular surfaces in specific contexts, which modulates their biology. At least in the case of BTN3A1, this dynamism drives the immunosuppression of αβ T cells or the activation of Vγ9Vδ2 T cells. Clearly, there is much to learn regarding the biology of BTN and BTNL molecules in the context of cancer, where they may represent intriguing immunotherapeutic targets that could potentially synergize with the current class of immune modulators in cancer. Here, we discuss our current understanding of BTN and BTNL biology, with a particular focus on BTN3A1, and potential therapeutic implications for cancer.

## 1. Introduction

The dissection of immune checkpoint biology has driven enormous advances in our understanding of how protective T cell activity is regulated in the tumor microenvironment [1]. Immune checkpoint molecules, e.g., PD-1, CTLA-4, B7-H3, B7-H4, drive the paralysis of protective T cells in tumor beds [2,3,4,5,6]. Consequently, translational research has focused heavily on developing monoclonal antibodies that target these molecules to restore protective T cell activity in cancer [5,6]. Indeed, several monoclonal antibodies targeting both PD-1 and CTLA-4 have been approved for the treatment of patients with cancer, where they have unquestionably improved the management of several malignancies [1]. However, the efficacy of Pembrolizumab and Ipilimumab in inducing clinically relevant response rates is limited in several solid epithelial malignancies. This includes advanced recurrent ovarian cancers [7] and advanced triple-negative breast cancer, where clinical efficacy is evident only in patients with a PD-L1 combined positive score of 10 or more [8]. Breast and serous ovarian carcinoma are indeed immunogenic and exert clinically relevant antitumor T cell pressure [9,10,11,12]. This immunogenicity is likely driven at least in part by genomic instability resulting from defects in homologous recombination repair machinery, which are frequently mutated in breast and ovarian cancers [13,14]. Despite the immunogenic nature of these malignancies, the current class of immune checkpoint immunotherapeutic approaches has demonstrated limited clinical benefit [7,8]. The ability of combination CTLA-4/PD-1 checkpoint therapies to restore protective T cell activity appears to be partially dependent on the presence of intraepithelial T cell and myeloid cell niches in ovarian cancer beds [9]. As suggested by others, the development of biomarkers characterizing the tumor-immune phenotype in high-grade serous ovarian cancer may identify patients with a greater likelihood to respond to PD1/PDL1 and CTLA4 blockade [15]. However, it is also critical to continue the interrogation of ovarian cancer immunobiology, as more therapeutically relevant, yet less well-characterized checkpoint molecules may be functioning as critical immunosuppressors in these ‘checkpoint resistant’ malignancies [16,17]. We therefore focus our discussion here on dissecting the known and potential roles of BTN and butyrophilin-like (BTNL) molecules in regulating as well as orchestrating T cell activity in breast and ovarian carcinomas. BTN and BTNL molecules appear to function as powerful immune modulators; however, the biology of this family of molecules has not been fully elucidated in the context of cancer. We propose that additional interrogations into the roles these molecules play in cancer progression as well as immune regulation will promote novel avenues to elevate antitumor immunity in these malignancies, which may prove to be more effective than the current class of checkpoint inhibitors in immunotherapy ‘refractory’ carcinomas. 

## 2. Butyrophilins: An Overview

BTN and BTNL molecules are a family of structurally related transmembrane glycoproteins that play important roles in regulating immune responses. In humans, they consist of 13 members; BTN1A1, BTN2A1, BTN2A2, BTN2A3, BTN3A1, BTN3A2, BTN3A3, BTNL2, BTNL3, BTNL8, BTNL9, BTNL10 (a pseudogene), and SKINT-like (SKINTL). The extracellular domains of BTN and BTNL molecules are structurally similar to the B7 family of costimulatory ligands; this includes PD-L1, B7-H3, and B7-H4, further suggesting an immunomodulatory role of these molecules. In limited studies to date, BTN/BTNLs have been shown to be involved in both activating and inhibiting T cell activity under homeostatic conditions as well as during pathological manifestations, such as cancer. Both BTN and BTNL molecules have a broad expression profile, with demonstrated surface expression on various immune cell subsets (Table 1) [18], as well as epithelial tissues such as the gut and some cancers; these include breast and ovarian carcinomas [17,19]. Additionally, polymorphisms of several BTN and BTNL molecules are associated with inflammatory diseases, including myositis and sarcoidosis [20,21,22,23], suggesting potential immunoregulatory roles. Indeed, the expression patterns of some BTN and BTNL molecules have been correlated with clinical outcome in patients with cancer [17,24,25]. However, knowledge about the extracellular and intracellular binding partners of many BTN/BTNL molecules and their mechanisms of signaling for any potential immunomodulatory function is still limited. As such, a comprehensive elucidation of the mechanistic activity of BTN/BTNL molecules potentially driving these associations in cancer has the potential to unleash a new class of cancer-directed immunotherapeutics.

## 3. Butyrophilins Regulate Protective Antitumor Immunity

BTN and BTNL molecules have gained the attention of tumor immunologists due to their ability to regulate T cell activity. Butyrophilin-like 2 (BTNL2) has the potential to suppress T cell activation through direct contact with the T cell surface, as chimeric BTNL2-Fc proteins have been demonstrated to impair T cell proliferation and cytokine production [26]. Likewise, murine BTNL2 has been demonstrated to function as a potent suppressor of the anti-tumor immune response. The antibody-mediated blockade of BTNL2 attenuated tumor progression in multiple in vivo murine tumor models, resulting in the prolonged survival of tumor-bearing mice [27]. Interestingly, studies utilizing murine T cells have shown an additional mechanism of BTNL2-mediated immune suppression where recombinant murine BTNL2 was found to promote T cell-intrinsic Foxp3 expression by eliciting the blockade of the Akt-mediated inactivation of Foxo1, therefore promoting a suppressive, regulatory-like T cell phenotype [28]. Thus, it is becoming evident that BTNL2 possesses an immunoregulatory role, which is likely critical in the progression of some malignancies, including colorectal cancer, where it has been demonstrated that BTNL-2 ablation in these mice reduced tumorigenesis by downregulating IL-22 production [29] (Table 2).

While the role of BTNL2 in protective immune response is becoming clearer, the functions of other BTNL molecules in anti-tumor immunity require further clarification. For example, BTNL8 is suggested to play an essential role in the priming of naïve T cells, however, it did not enhance the production of memory T cells [39]. This suggests BTNL8 may be advantageous in cancer immunotherapy by potentially provoking polyclonal ‘effector-like’ T cell responses, which have been associated with better outcomes for some cancer patients. Moreover, BTNL8 has also recently been reported to drive the activation of subsets of gut-resident γδ T cells through cell surface interactions with BTNL3 [40]. While lacking mechanistic investigation, both BTNL3 and BTNL8 recombinant proteins appear to drive the differential regulation of interferon-gamma secretion from CD3^+^ T cells (e.g., rndsystems.org, accessed 3 May 2023.), suggesting potential contextual implications for their impact on both αβ and γδ T cell functionality. While these molecules are widely expressed in cancer, their impact in this setting remains unknown. Of the butyrophilin-like molecules, BTNL9 remains the most poorly characterized in humans. However, its expression has been positively associated with higher immune infiltration and survival in a cohort of patients with lung adenocarcinoma [38] (Table 1), suggesting a possible immune co-stimulatory function. Mechanistically, it was demonstrated that mutant p53 is a significant factor contributing to decreased BTNL9 expression in patients with breast cancer. Intriguingly, in a 2021 study, Mo et al. revealed the impact of BTNL9 expression on the progression of breast cancer through cancer cell-intrinsic regulation of the cell cycle. In this study, the elevated expression of BTNL9 blocked breast cancer cells in G2/M via P53/CDC25C and P53/GADD45 pathways [41], suggesting that BTNL9 could be a promising biomarker for the early detection of breast cancer, thereby enabling timely and effective treatment. Thus, while BTNL molecules may most logically play a role in immune regulation due to their homology to B7 family members, the potential immune-independent regulation of cancer progression by BTNL molecules may represent an intriguing area of investigation.

The activity of BTN molecules also drives immune regulatory activity. BTN2A2 appears to dampen dysregulated T cell activity associated with autoimmunity, as the exogenous administration of BTN2A2-IgG2a Fc proteins ameliorated the pathogenicity of a disease modeling rheumatoid arthritis in mice through the impairment of T cell proliferation and production of Th1/Th17 cytokines [42]. Additionally, BTN3A1, while currently appreciated as an activator of Vγ9Vδ2 T cells [40,43,44,45], was described over a decade ago as a suppressor of αβ T cells. In 2010, BTN3A1 was found to be widely expressed on immunosuppressive cells found within the ovarian tumor microenvironment and to function as a suppressor of T cell activity by leveraging in vitro studies utilizing K562 cells ‘presenting’ BTN3A1 on their surface [16]. Supportive of the immunomodulatory activity of BTN3A1, the concentration of soluble BTN3A1 found in the plasma of metastatic clear cell renal carcinoma patients was found to predict therapeutic responses to the checkpoint immunotherapeutic nivolumab [31]. Intriguingly, while the BTN3A family likely has shared functional activity in suppressing αβ T cells due to the nearly identical homology of their extracellular domains, BTN3A3 has also been demonstrated to possess an immunomodulatory function independent of T cells. In fact, xenograft models of breast cancer expressing tumor cell surface BTN3A3 modulated the function of macrophages to promote the acquisition of a cancer stem cell (CSC)-like phenotype [32]. Mechanistically, macrophages engaged BTN3A3 through surface expression of the C-type lectin, LSECtin, to promote this phenotype. Either macrophage-specific ablation of LSECtin or silencing of BTN3A3 in breast cancer cells decreased CSC frequency and tumor growth. BTN2A1, furthermore, has been found to engage CD209 (also known as DC-SIGN) on the surface of immature monocytes through high-mannose oligosaccharides [46]. More recently, BTN3A1 has been shown to engage CD45 on the surface of αβ T cells through an N-linked glycan-dependent mechanism [17]. Curiously, nearly all BTN and BTNL molecules are predicted to be post-translationally modified with glycosylation motifs [47], suggesting these carbohydrate moieties may be important in driving the biology associated with these molecules. Collectively, previous work investigating BTN/BTNL molecules clearly demonstrates their potential to modulate immune cell activity. Clarifying the roles of these molecules in cancer and, subsequently, their potential to function as immunotherapeutic targets may drive the development of more effective cancer immunotherapies, as discussed in the case of BTN3A1 below.

## 4. BTN3A1 Impairs Tumor-Specific αβ T Cells in Cancer

**Figure 1:**
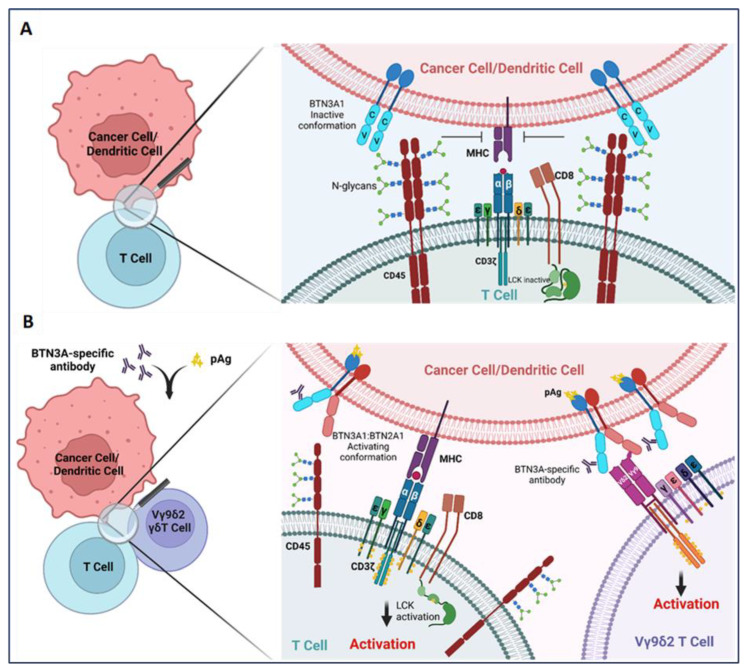
BTN3A1 transformation from an immunosuppressive to an immunostimulatory molecule upon sensing pAgs or anti-BTN3A-specific antibodies. (**A**) BTN3A1 engagement with N-glycans of CD45 inhibits segregation of N-glycosylated CD45 from the immune synapse on the surface of αβ T cells and prevents the activation of the tumor-reactive T cells; (**B**) Anti-BTN3A antibodies or pAgs sensed by BTN3A1 drive BTN3A1:BTN2A1 interactions; BTN2A1 binds to the Vγ9 TCR chain and promotes Vγ9Vδ2 subset of γδ T cell activation. Transformation of BTN3A1 from an immunosuppressive molecule (through engagement with N-glycosylated CD45) for tumor antigen-specific T cells to an immunostimulatory molecule also rescues αβ T cell anti-tumor activity.

BTN3A1 is most widely appreciated to function as an activator of the Vγ9Vδ2 subset of γδ T cells, in concert with BTN2A1 [40,43,44,45,48]. Intriguingly, however, it has been reported that BTN3A1 also functions as a regulator of αβ T cells [16,17]. In a mechanistic interrogation of the impact of BTN3A1 on antitumor immunity, Payne et al. found the dysregulated expression of this molecule in high-grade serous ovarian cancer (HGSOC) and triple-negative breast cancer [17,25]. While dysregulated expression has been observed on the surface of malignant cells and infiltrating leukocytes—most notably dendritic cells—in both malignancies, the mechanisms driving aberrant overexpression of BTN3A1 in cancer remain unknown [16,17]. However, this dysregulated expression is likely a result of aberrant NLRC5 activity in these malignancies, as NLRC5 was recently found in the transcription of *BTN3A* genes through an atypical SXY module within the major histocompatibility complex [49]. Importantly, this dysregulated expression of BTN3A1 was found to be inversely correlated with HGSOC patient survival [17]. Critically, in a mechanism which appears to be distinct from its ability to activate Vγ9Vδ2 T cells, recombinant BTN3A1-Fc chimeric proteins were demonstrated to impair the phosphorylation of activating tyrosine residues in signaling molecules proximal to the αβ T cell receptor [17], while K32 cells ectopically expressing BTN3A1 impaired the proliferative potential of purified αβ T cells in vitro [16]. By leveraging novel transgenic mice in which BTN3A1 was specifically expressed in dendritic cells, Payne et al. demonstrated an elevated malignant progression which paralleled the decreased infiltration and effector activity of αβ T cells in the setting of ovarian cancer. Targeting BTN3A1 in vivo using CTX-2026, a unique fully human aglycosylated monoclonal antibody delayed the progression of ovarian cancer in both syngenic and xenograft models through the elicitation of more robust αβ T cell responses. Additionally, targeting BTN3A1 with CTX2026 impaired malignant progression more robustly than the PD-1 inhibitor, Nivolumab. Mechanistically, it was found that BTN3A1 engages CD45 of the surface of αβ T cells through an N-linked glycosylation-dependent mechanism independent of BTN2A1; this engagement was found to physically impede the formation of the immune synapse through impairing the segregation of CD45 from the T cell receptor [17,50] (Figure 1). This study underscores the importance of BTN3A1 in blunting protective antitumor T cell activity in the setting of cancer, and lays bare the proposition that targeting this immunosuppressive pathway may be more effective in unleashing protective immunity than the blockade of PD-1/PD-L1 in certain malignancies.

## 5. Vγ9Vδ2 T Cells Are Regulated by BTN3A1 and Are Dynamic Immune Effectors in Cancer

BTN and BTNL molecules are also critical regulators of γδ T cells in humans. Unlike conventional αβ T cells, γδ T cells do not require antigen presentation by the major histocompatibility complex cells, which comprise 1% to 10% of all T cells in humans [51,52], and their accumulation into high-grade serous ovarian cancer beds is associated with better overall survival [17]. These cells express a combination of Vγ TCR chain (Vγ1 2, 3, 4, 5, 8, 9, and 11) which pair with one of four Vδ TCR chains (Vδ1, 2, 3, and 5) [52,53]. 

Vγ9Vδ2 T cells account for ~5% of all peripheral T cells and are recognized for their ability to rapidly degranulate upon their activation to kill cancer cells [54]. Low levels of Vγ9Vδ2 T cells in breast cancer patients (compared with healthy individuals) were found to be correlated with a higher T stage, a measure of the extent of the tumor [55,56], suggesting Vγ9Vδ2 T cells may be critical antitumor effectors in this malignancy. BTN3A1 has been the most extensively studied BTN family member in relation to γδ T cells. For more than a decade, BTN3A1 has been recognized for its ability to activate Vγ9Vδ2 T cells. It has become appreciated over time that phosphoantigen (pAg) ‘sensing’ by the intracellular B30.2 domain of BTN3A1 is critical in the activation of Vγ9Vδ2 T cells [57,58]. pAgs accumulate in the cytosol under cellular stress, including infection and malignant transformation, as well as through the activity of aminobisphosphonates [59,60]. However, early attempts to indisputably demonstrate the sufficiency of BTN3A1 in activating Vγ9Vδ2 T cells through the direct binding of BTN3A1 to either the Vγ9 chain or the Vδ2 chain of the TCR failed [43,44,61]. The requirement of additional genes on chromosome 6 for the activation of Vγ9Vδ2 T cells was then identified [45]. In recent ground-breaking work by Rigau et al., a genome-wide CRISPR/Cas9 screen revealed *BTN2A1* as the essential additional gene required for driving Vγ9Vδ2 T cell activation. pAg sensing by BTN3A1 thus was found to promote cell surface concurrent BTN3A1:BTN2A1 interactions, which facilitated the direct binding of BTN2A1 to the Vγ9Vδ2 TCR in a glycan-independent manner [48]. Additionally, a recent investigation suggested that the BTN3A1 IgV domain may also mediate indirect interactions with the Vγ9Vδ2 TCR [62]. Critically, CRISPR/Cas9-mediated ablation of BTN2A1 did not impair the suppressive potential of BTN3A1 against αβ T cells in the setting of ovarian cancer [17], suggesting that the particular dynamics of BTN3A1:BT2A1 interactions can dictate either the activation of Vγ9Vδ2 T cells, or the suppression of αβ T cells. This was further supported by the observed loss of BTN3A1-mediated suppression against αβ T cells upon treatment of BTN3A1/BTN2A1 expressing ‘artificial antigen-presenting cells’ with the aminobisphosphonate Zoledronate [17].

Due to their general accumulation in epithelial tumor beds, as well as the ease of their ex vivo expansion, Vγ9Vδ2 T cells are being broadly leveraged as an immunotherapeutic for cancer. Initially, cancer immunotherapy leveraging Vγ9Vδ2 T cells focused on utilizing aminobisphosphonates (e.g., pamidronate and zoledronate), which inhibit an enzyme in the mevalonate pathway, leading to the accumulation of phosphoantigens, for in situ activation or ex vivo expansion followed by cellular infusion in a variety of cancers, as reviewed elsewhere [63]. More recently, however, specific antibodies targeting the extracellular domain of BTN3A1 promote the activation of Vγ9Vδ2 T cells with similar efficacy to aminobisphosphonates. Two mouse antibodies, clones 7.2 and 20.1, have been reported to induce Vγ9Vδ2 T cell activation and degranulation in a BTN3A1-dependent manner, while the fully human clone CTX-2026 was reported to activate these cells with equivalent efficacy. Both 20.1 and CTX2026 share an epitope within the distal IgV domain of the BTN3A1 extracellular domain [17,64]. These two clones delay malignant progression through promoting the cytotoxic potential of adoptively transferred Vγ9Vδ2 T cells against several tumor types, including ovarian cancer, in preclinical xenograft animal studies. While the mechanism for these effects is not clear, pAg sensing or antibody-induced crosslinking of BTN3A1 and BTN2A1 is a likely explanation [17,62]. The engagement of all BTN3A isoforms by specific antibodies may be more effective than the use of bisphosphonates in B30.2-independent activation of cytotoxic γδ T cells.

In a critical advance in targeting BTN3A1 in the setting of cancer, Imcheck Therapeutics has developed a BTN3A-specific antibody, ICT01, a humanized version of clone 7.2 that recognizes an epitope within BTN3A1 which overlaps with clone 20.1. An ongoing phaseI/IIa trial (NCT05307874—EVICTION-2) testing the efficacy of ICT01 with low-dose IL-2 against advanced-stage solid tumors (including breast, ovarian, and colorectal cancer) has demonstrated the ability to reproducibly induce the activation and expansion of not onlyVγ9Vδ2 T cells, but also CD8^+^ T cells in 6/6 patients with relapsed/refractory ovarian and colorectal cancer. No dose-limiting toxicities have been observed. These preliminary clinical data demonstrating the expansion of both γδ and αβ T cells truly underscores the potential dual effect of targeting BTN3A in malignancies to promote the activity of classical αβ T cells in addition to the better-characterized activation of Vγ9Vδ2 T cells, which may therefore drive combinatorial clinical benefit.

Furthermore, Imcheck Therapeutics is conducting a Phase 1/2a clinical trial (NCT04243499) to evaluate the safety and efficacy of ICT01 in combination with pembrolizumab in patients with advanced or metastatic solid tumors. The study is designed to assess the safety, pharmacokinetics, pharmacodynamics, and preliminary efficacy of the combination therapy in patients with advanced or metastatic solid tumors. Preliminary data from this study indicate that higher levels of tumor infiltration by γδ T cells, CD8^+^ αβ T cells, and natural killer cells are linked to better clinical outcomes. These data suggest combinatorial approaches leveraging the current classes of immunotherapies with butyrophilin modulators may promote a more effective setting of protective ‘immune synergy’ in cancer beds. 

## 6. Conclusions and Future Perspectives

It is obvious that elevating the immunotherapeutic tools available to target breast and ovarian cancers could potentially provide more effective and viable options to impair malignant progression of these diseases. Here, we discussed our current understanding of the impact of BTN and BTNL molecules in modulating immune activity and cancer progression. Additional preclinical and clinical interrogations to illuminate the impact of targeting BTN3A1 and/or BTN2A1 to support both αβ and γδ protective T cell activity is critically needed, most notably in γδ T cell enriched tumors, such as breast and ovarian carcinomas [55,56]. Indeed, elegant approaches are being utilized to improve the anti-cancer therapeutic efficacy of Vγ9Vδ2 T cells, including new BTN3A antibodies as well as the development of novel synthetic tools (e.g., chimeric antigen receptor-γδ T constructs and novel Vγ9Vδ2 TCR-tumor antigen bispecific molecules) [63]. Furthermore, the identification of BTN and BTNL molecule expression patterns in dormant cancer cells may offer creative avenues to eliminate residual disease before recurrence, which is the main driver of disease-related mortality in breast cancer [65,66,67].

In addition to understanding how other BTN and BTNL molecules regulate αβ T cells in the tumor microenvironment, as well as their ‘targetability’ using antibodies or other synthetics, a critical question is to what extent BTN and/or BTNL molecules regulate subsets of γδ T cells other than the Vγ9Vδ2 subset in human cancers. For instance, Vδ1 T cells have been observed in several malignancies and at frequencies several times higher than Vγ9Vδ2 T cells; it has been suggested that this cellular population has either a cytotoxic or, conversely, an immunosuppressive role [63,68]. It is fundamentally clear that interrogating the Vδ1 subset of γδ T cells will further advance our knowledge of the immunobiology of cancer. Interestingly, butyrophilins may also play a critical role in the regulation of this less well-characterized subset of γδ T cells. As Vδ1 T cells are mainly found in the gut, where they are critical for maintaining tissue homeostasis [69,70], complexes of BTNL3 and BTNL8 on the surface of intestinal epithelial cells are critical for at least the activation of Vγ4Vδ1 T cells [40,71]. How other Vδ1 T cells utilizing diverse Vγ chains may potentially respond to BTN/BTNL activity as well their potential heterogeneous activity in cancer beds remains unknown. Further interrogation of these cells and their likely regulation by BTN/BTNL molecules will unquestionably deepen our understanding of cancer immunology and open possibilities for novel immunotherapies. 

Here, we put forth that butyrophilin biology in the context of cancer generally remains unknown. We have gained a better appreciation of the impact of the dynamism of butyrophilin and butyrophilin-like molecules on the activity of γδ and αβ T cell activity, which has driven clinical interrogations of the efficacy of targeting BTN3A in a variety of malignancies. The next critical step is to gain a greater mechanistic understanding of the regulation of BTN and BTNL molecules in cancer, as well as any associated implications for T cell biology, including the poorly understood Vδ1 T cell subset. These efforts will not only unveil novel perspectives regarding how the association of immune regulatory molecules on the cell surface modulate immune effector function, but also provide novel insights into the orchestration of immune suppressive programs in cancer.

## Figures and Tables

**Table 1:** The expression profile of the Butyrophilin family on different immune cell subsets.

Gene	Predicted Location	The Expression on Immune Cell Subsets
BTN1A1	Membrane	Memory B-cell, Naive B-cell, Non-classical monocyte, Eosinophil, MAIT T cells
BTN2A1	Intracellular, membrane	Neutrophil, Basophil, Eosinophil, T-reg, Memory CD8 T-cell, γδ T-cell, Naive CD8 T-cell, NK-cell, MAIT T-cell, Memory CD4 T-cell, Classical monocyte, Plasmacytoid DC, Non-classical monocyte, Naive CD4 T-cell, Memory B-cell, Myeloid DC, Total PBMC, Naive B-cell, Intermediate monocytes
BTN2A2	Intracellular, membrane	Naive B-cell, Memory B-cell, Myeloid DC, Intermediate monocyte, Classical monocyte, Neutrophil, T-reg, Plasmacytoid DC, NK-cell, Total PBMC, Non-classical monocyte, γδ T-cell, MAIT T-cell, Memory CD8 T-cell, Naive CD8 T-cell, Memory CD4 T-cell, Naive CD4 T-cell, Eosinophil, Basophil
BTN3A1	Intracellular, membrane	Basophil, γδ T-cell, Memory CD8 T-cell, NK-cell, T-reg, Naive CD8 T-cell, Eosinophil, Total PBMC, Memory CD4 T-cell, Naive CD4 T-cell, Neutrophil, Memory B-cell, Non-classical monocyte, Intermediate monocyte, Classical monocyte, Naive B-cell, Myeloid DC, Plasmacytoid DC
BTN3A2	Intracellular, membrane	γδ T-cell, Memory CD8 T-cell, T-reg, Naive CD8 T-cell, NK-cell, MAIT T-cell, Naive CD4 T-cell, Memory CD4 T-cell, Total PBMC, Basophil, Memory B-cell, Eosinophil, Naive B-cell, Non-classical monocyte, Neutrophil, Intermediate monocyte, Classical monocyte, Myeloid DC, Plasmacytoid DC
BTN3A3	Intracellular, membrane	Memory CD8 T-cell, γδ T-cell, Basophil, Naive CD8 T-cell, T-reg, NK-cell, MAIT T-cell, Memory CD4 T-cell, Naive CD4 T-cell, Total PBMC, Non-classical monocyte, Intermediate monocyte, Memory B-cell, Classical monocyte, Naive B-cell, Eosinophil, Myeloid DC, Neutrophil, Plasmacytoid DC
BTNL2	Intracellular, membrane	Not detected in Immune cells
BTNL3	Membrane	Neutrophils
BTNL8	Membrane	Neutrophil, Eosinophil
BTNL9	Membrane	Memory B-cell, Naive B-cell, Basophil, Neutrophil, Eosinophil, Classical monocyte

Courtesy of Human Protein Atlas, www.proteinatlas.org, accessed on 3 May 2023, [18]. The table provides insight into the transcriptional profile of butyrophilins and butyrophilin-like molecules in human immune cell subsets. Cell types with a normalized transcripts per million (nTPM) value ≥0.2 are included in the table. Abbreviations: BTN, butyrophilins; BTNL, butyrophilin-like; DC, dendritic cells; PBMC, peripheral blood mononuclear cells.

**Table 2:** Butyrophilin family and its associations in various malignancies.

Gene	Association in Different Cancer Type	Reference
BTN2A1	Renal cell carcinoma	[30]
BTN3A1	Ovarian cancer, Breast cancer, bladder cancer, pancreatic ductal adenocarcinoma and renal cell carcinoma	[17,31,32,33,34]
BTN3A3	Ovarian cancer, Breast cancer, non-small cell lung cancer	[32,35,36]
Butyrophilin-like 2 (BTNL2)	Prostate cancer, colorectal cancer	[29,37]
Butyrophilin-like 9 (BTNL9)	Lung cancer	[38]

This study utilized a systematic literature review methodology to identify and analyze research articles on BTN and BTNL molecules in the last 20 years. The study used PubMed as the primary search tool and the search was conducted using the following search terms: BTN, BTNL basic biology, expression profile in immune cells and in cancer, and impact on γδ and αβ T cells and functional impact across different human malignancies. Articles published in English within the last 20 years met this study’s inclusion criteria. The study did not involve any human or animal subjects, and all data were collected from publicly available sources. Abbreviations: BTN, butyrophilins; BTNL, butyrophilin-like.

## Data Availability

The data presented in this study are openly available in The Human Protein Atlas at 10.1126/science.aax9198, reference number [18].
